# Sequence Diversity and Expression Profiles of T Cell Receptor Beta Chain Constant Genes *TRBC1* and *TRBC2* in Canine Lymphoid Tumour Cell Lines and Normal Lymphocytes

**DOI:** 10.1111/vco.70003

**Published:** 2025-07-19

**Authors:** Marek Pieczka, Leszek Moniakowski, Aleksandra Studzińska, Dominika Kubiak‐Nowak, Aleksandra Pawlak, Arkadiusz Miazek

**Affiliations:** ^1^ Department of Biochemistry and Molecular Biology Wroclaw University of Environmental and Life Sciences Wroclaw Poland; ^2^ Department of Surgery Wroclaw University of Environmental and Life Sciences Wroclaw Poland; ^3^ Department of Pharmacology Wroclaw University of Environmental and Life Sciences Wroclaw Poland

**Keywords:** canine lymphoma, cross‐lineage rearrangement, immunotherapy, TRBC

## Abstract

The subtle sequence diversity and mutually exclusive expression patterns of T cell receptor beta chain constant genes, *TRBC1* and *TRBC2*, in mature human T cells, provide the basis for immune‐targeting strategies designed to eliminate clonally expanded malignant T cells while sparing a subset of normal T cells capable of maintaining immunocompetence. The evolutionarily conserved gene arrangement and regulation of *TRBC* loci in mammals make these genes attractive targets for translational immune‐targeting strategies in companion species, including dogs. However, available *TRBC* sequence data relevant to common dog breeds remains limited. In this study, we investigated the sequence diversity and mRNA expression profiles of canine *TRBC1* and *TRBC2* genes in peripheral blood mononuclear cell (PBMC) samples representing 14 different dog breeds, and in six established canine haematopoietic cell lines of both T‐cell and non‐T‐cell origin (i.e., B and NK cell lines). Our analysis uncovered a previously unreported variation in the *TRBC1* sequence encoding the transmembrane region but found no sequence diversity in the extracellular domain of TRBC1 and TRBC2. A nearly equal mRNA expression of TRBC1 and TRBC2 was consistently observed in bulk samples of canine PBMCs across all breeds, in contrast to canine cell lines, which exhibited a more skewed expression profile. Unexpectedly, germline mRNA expression of *TRBC* was present in some (i.e., CLB70, GL1) but not other (i.e., CLBL1) canine cell lines of B cell origin. In conclusion, our findings indicate that the fully conserved amino acid sequence in the extracellular domain of canine TCR beta chain variants presents a challenge for the development of differential therapeutic antibodies. Additionally, the presence of germline *TRBC* transcripts in certain canine B‐cell neoplasms, but not others, may provide additional insights into the developmental stages from which these neoplasms originate.

Human and canine T cell lymphomas exhibit numerous parallels, including comparable incidence, clinical presentation, histological and molecular characteristics, response to treatment, and prognosis [1, 2]. As with B cell lymphoma, no consistently expressed, tumour‐specific cell surface markers in human or canine T cell lymphomas are available for immunotherapy [3]. Moreover, in contrast to the B‐cell compartment, depleting the entire T cell compartment with monoclonal antibodies is immunosuppressive or toxic, making it unsuitable as an effective anti‐tumour therapy [4].

Recent efforts to develop immunotherapies for human T cell malignancies have focused on the mutually exclusive expression of T cell receptor beta chain constant variants 1 and 2 (*TRBC1* and *TRBC2* genes) [5]. In humans, minor amino acid differences in the extracellular constant domain of TRBC1 and TRBC2 have been sufficient to generate variant‐specific antibodies. These antibodies facilitate the diagnosis of T cell lymphomas via flow cytometry and enable experimental CAR T cell therapies aimed at selectively depleting malignant T cells while preserving part of the normal T cell population [6, 7]. However, whether this approach can be applied to canine T cell malignancies remains unclear due to limited and conflicting sequencing data on the *TRBC* locus across different dog breeds.

In most mammals, the *TRB* locus, which encodes the T cell receptor beta chain (*TRB*), consists of 34–77 *TRBV* (variable) gene segments located upstream of two (or three, in artiodactyls) [8] *TRBD‐J‐C* clusters, each composed of one *TRBD* (diversity) gene, six to eight *TRBJ* (joining) genes, and one *TRBC* (constant) gene [9]. The *TRBC* gene encodes a constant domain, connecting region, transmembrane region, and short cytoplasmic tail. Between human TRBC1 and TRBC2, only four amino acid differences have been reported [10]. The canine *TRBC* locus was initially reported in 2009 as containing a single *TRBD‐J‐C* cluster [11]. However, a 2012 revision demonstrated that it more closely parallels the human and murine loci, each comprising two *TRBD‐J‐C* clusters [12]. This later study identified a single amino acid variation between the constant domains of canine *TRBC1* and *TRBC2*, with a serine codon at position p.15 for *TRBC1* and a proline codon for *TRBC2*. However, other studies have not independently confirmed this variation [13]. Based on the reports by Mineccia et al. [12] and Martin, J et al. [14] the IMGT database currently lists two canine *TRBC1* alleles (TRBC1*01 and TRBC1*02) and one *TRBC2* allele (TRBC2*01) differing by their respective 3′UTR sequences and single amino acid substitutions in coding sequence listed in Table 1. To independently confirm the reported sequence variations across various dog breeds and to identify any additional ones, we reverse‐transcribed and PCR‐amplified full‐length *TRBC1* and *TRBC2* mRNA transcripts from 20 PBMC samples representing 14 different dog breeds (Table 2) (see Supporting Information and Methods for RT‐PCR conditions and primer sequences). We then subjected the amplification products to Sanger DNA sequencing and used standard sequence alignment tools for sequence comparisons. These sequence data have been submitted to the GenBank database under accession number PRJNA1098592. Our analyses revealed that both *TRBC1* and *TRBC2* have a serine codon at position p.15 in all samples analysed, and no other variations in the extracellular region of canine *TRBC* were found (Table 1). Therefore, the proline residue at position p.15 of TRBC2*01, reported by Mineccia et al. [12], represents a rare polymorphism whose calculated frequency in the canine population is no greater than 9,53 × 10^‐7 (see Supporting Information and Methods). In the transmembrane domain of TRBC, we confirmed a previously reported variation at position p.158 (TRBC1‐valine/TRBC2‐isoleucine) and identified a novel, in‐frame two‐codon deletion at position p.163/164 (encoding alanine and lysine residues, respectively). This deletion was homozygous in 65% (13/20), heterozygous in 25% (5/20), and absent in 10% (2/20) of *TRBC1* PBMC samples analysed, with a calculated allelic frequency equal to 0.775 (Tables 1 and 2). Interestingly, this deletion reduces the total positive charge of the TRBC1 C‐terminal region that otherwise contains a stretch of 3 positively charged lysine and arginine residues, forming an endoplasmic reticulum (ER) retention motif [15]. The above‐mentioned TRBC1 deletion variant, termed by us as TRBC1Δ, contains only a partial ER retention motif. The *TRBC1Δ* mRNA expression was also detected in the CLB70 B cell leukaemia cell line but not in the PER‐VAS T cell lymphoma cell line, which exclusively expressed TRBC2 (Table 2). Whether this partial ER retention motif is sufficient for proper T cell receptor assembly and transport to the cell surface remains to be experimentally determined and is beyond the scope of this report.

**TABLE 1:** Canine *TRBC* alleles.

TRBC allele	Source	Accession number	p. 15^a^ S/P	p. 158 V/I	p. 163–164 deletion	c.535–564^b^ 3′UTR	Frequency in population
TRBC1*01	IMGT	IMGT000005	S	I	−	C2^c^	Not observed
TRBC1*02	IMGT	HE653929	S	V	−	C1^d^	0.225
TRBC1Δ	This study	SRR28607731	S	V	+	C1	0.775
TRBC2*01	IMGT	HE653929	P	I	−	C2	Not observed
TRBC2	This study	SRR28607730	S	I	−	C2	1

^a^ Position in protein sequence of TRBC with single letter amino acid description of variants.

^b^ Position in mRNA transcript of TRBC starting from the beginning of exon 1.

^c^ 3′UTR sequence adjacent to the stop codon of TRBC characteristic of TRBC2 transcripts [12]: (5′UGAGACCAGCUCCAAAAGUGCAUCCUGAGAUGA).

^d^ 3′UTR sequence adjacent to the stop codon of TRBC characteristic of TRBC1 transcript [12] (5′UGAAACUAGUUCAGAGGCAGAGCCAGCAGCUUC).

**TABLE 2:** Expression of *TRBC* alleles within different dog breeds and canine haematopoietic cell lines.

Breed	Sample *n*°	TRBC1*02	*TRBC1Δ*	*TRBC2*
Scrub	6	−	+	+
	8	−	+	+
	19	−	+	+
	27	−	+	+
	32	−	+	+
Beagle	23	−	+	+
	24	+	−	+
	25	−	+	+
Akita	5	+	−	+
Alaskan malamute	22	+	−	+
AmStaff	31	+	−	+
Australian Shepherd	20	+	−	+
Boxer	18	+	−	+
Border collie	26	+	−	+
Cane corso	17	−	+	+
German shepherd	29	−	+	+
Pomeranian	30	−	+	+
Pudel toy	28	−	+	+
WHWT	7	−	+	+
Yorkshire terrier	21	−	+	+
CLB70	N/A	−	+	+
CNK89	N/A	No data	No data	+
GL1	N/A	No data	No data	+
PER‐VAS	N/A	No data	No data	+

*Note:* (+)/(−) the presence or absence of a given trait.

To investigate whether *TRBC1* and *TRBC2* genes are equally expressed in normal canine peripheral T cells, we used *TRBC* variant‐specific primers to perform real‐time PCR on cDNA templates from five PBMC samples representing different dog breeds (see Supporting Information and Methods for PCR specificity assessment and detailed primer sequence data). Consistent with findings in other mammals, we observed a roughly equal 50/50 expression ratio of *TRBC1* and *TRBC2* cDNA independent of breed (Figure 1A). However, when we applied this technique to cDNA from six canine lymphoid cell lines, we observed unequal *TRBC* expression patterns in all but one (CL1‐ T‐lymphoblastoid cell line derived from malignant lymphoma). Of note, *TRBC* was expressed in B‐cell lines (GL1, CLB70) except CLBL1 and NK‐cell line CNK89 (Figure 1B). To further investigate the identity of *TRBC* transcripts expressed in selected canine cell lines, we performed 5′ rapid amplification of cDNA ends (5′RACE) followed by DNA sequencing. As shown in Figure 2 and Table S3, a productive V, D, J rearrangement of *TRB* genes was only detected in the PER‐VAS T‐cell line. In contrast, all other tested cell lines exclusively expressed germline *TRB* transcripts.

**FIGURE 1:**
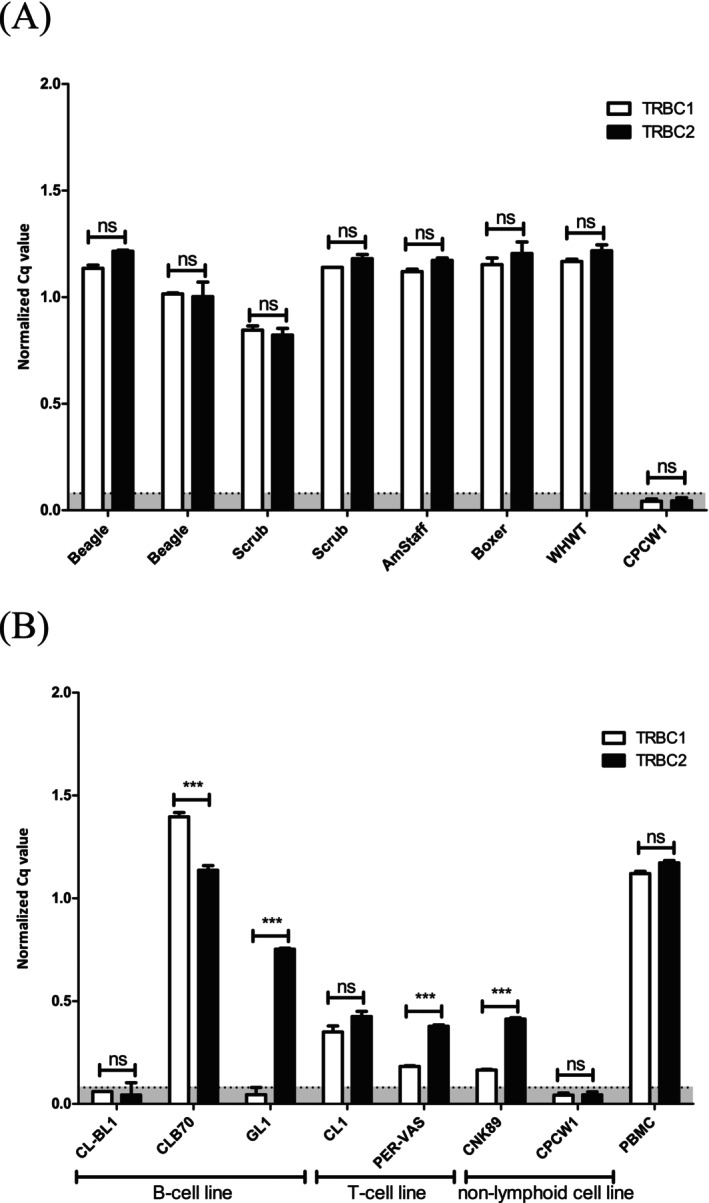
Real‐time RT‐PCR assessment of *TRBC1* vs. *TRBC2* expression levels in control epithelial cell line CPCW1 (Zacharski et al. Unpublished), peripheral blood leukocytes (PBMC) of dogs representing various breeds (A), and in canine lymphoid cell lines (B). Cq values were normalised to *GAPDH*. The error bars depict six technical repeats. For both (A) and (B) symbol * indicates statistical significance, whereas *** indicates a strong statistical significance, * indicates a weak statistical significance, and ‘ns' means a lack of statistical significance. The horizontal dashed line and the shaded grey area below it indicate the threshold for background gene expression.

**FIGURE 2:**
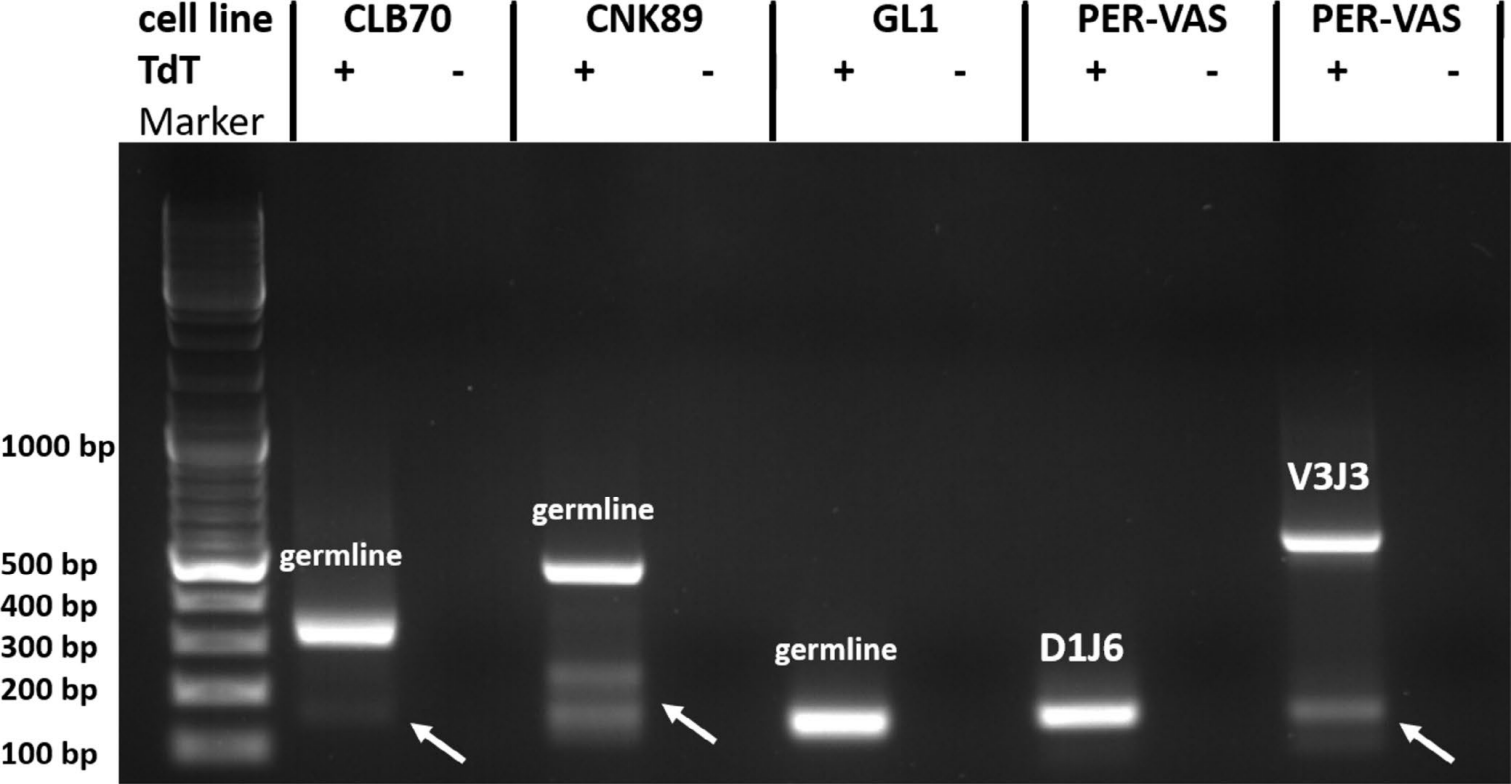
5′‐rapid amplification of cDNA ends (5′RACE) on cDNA samples from indicated cell lines in the presence (+) or absence (−) of TdT poly‐A tailing. The double‐stranded DNA ladder (Marker) in base pairs (bp) is run in the first lane. PER‐VAS cDNA generated two kinds of 5′RACE products, which were amplified using different primer annealing temperatures (see Supporting Information and Methods). Sequences of identified germline and V, D, J or D, J transcripts are shown in Table S3. Arrows indicate additional 5′RACE products consistently present in analysed cell lines.

Germline *TRB* transcripts appear among the first lineage differentiation steps during T cell development and are linked to chromatin remodelling, enabling the accessibility of the recombinase apparatus to the TCR beta locus. These transcripts are promoted by cis‐acting Dβ1 promoter and Eβ enhancer elements in a cell lineage specific manner at the pro‐T cell stage of thymocyte maturation, where the cell fate is not yet fully determined [16]. Numerous reports have found germline *TRB* transcripts in B cell lines derived from immature precursors and even ex vivo isolated tonsillar B cells, albeit at very low levels [17]. It has also been reported that germline *TRB* transcripts continue to be expressed from an unrearranged and/or nonproductively rearranged allele throughout T cell maturation [18].

The 5′RACE analysis of PER‐VAS cell line provided evidence that partially rearranged germline *D1J6‐TRBC* and productively rearranged V3J1*TRBC* alleles are coexpressed (Figure 2). Similar coexpression of different germline *TRBC* transcripts identified by 5′ RACE were observed in CLB70 and CNK89 cell lines but not GL1 cell line, which transcribed only a single germline *TRBC2* transcript (Figures 1 and 2). The above‐described observations are reminiscent of reported germline *TRB* expression from the hypomethylated *TRB* locus in human B‐cell non‐Hodgkin's lymphoma cell lines [19] and precursor type acute B lymphocytic leukaemia (B‐ALL) [20] arising from incompletely committed B cell precursors. The molecular basis of germline *TRBC* expression in immature B cell neoplasms is not fully understood but may reflect an incomplete B cell lineage commitment due to insufficient expression and/or the presence of inactivating mutations of PAX5 transcription factor [21]. In this context, we observed significantly higher levels of *PAX5* transcripts in CLBL1 cell line, which represents B‐cell plasmacytoid lymphoma (derived from terminally differentiated B cells) than in GL1 acute B‐cell leukaemia and CLB70 chronic B cell leukaemia cell lines, both representing immature B cell precursor neoplasms (Pieczka and Miazek, unpublished observations).

In summary, the *TRBC* sequence analysis presented in this study identified a novel deletion variant of the *TRBC1* allele, termed *TRBC1Δ*, which carries a two‐codon deletion in the transmembrane region. However, the extracellular domains of TRBC1 and TRBC2 were identical in all analysed samples.

Although additional, currently unidentified mutations may exist in *TRBC* genes, their low prevalence likely does not warrant the development of targeted antibodies for immunotherapy of canine T cell malignancies. As such, while the approach cannot be deemed entirely unfeasible, it appears to lack economic justification.

Additionally, we report the expression of germline *TRBC* transcripts in canine acute B‐cell leukaemia (CLB70), chronic B‐cell leukaemia (GL1), and NK‐cell lymphoma (CNK89) cell lines, whereas *TRBC* expression was absent in the terminally differentiated plasmacytoid B‐cell lymphoma cell line (CLBL1). The molecular analysis of *TRBC* rearrangement and expression status in canine haematopoietic cell lines, facilitated by the tools described in this study, may offer further insights into the developmental origins of these neoplasms.

## Conflicts of Interest

The authors declare no conflicts of interest.

## Supporting information


**Table S1.** Primers used for PCR and 5′RACE.
**Table S2.** Sequences of synthetic *TRBC1* and *TRBC2* DNA fragments with their length.
**Table S3.** 5′UTR sequences (grey colour), V (variable), D (diversity) and J (joining) regions (green colour), and first coding exon of TRBC gene (blue colour).
**Figure S1.** Schematic representation of the TRBC1 and TRBC2 loci with positions of PCR primers. Gene‐specific primers (UTRC1 and UTRC2) are positioned in 3′untranslated regions (grey boxes) whereas primers common for both TRBC1 and TRBC2 isoforms (cTRBC_F, GS1, GS2, and GS3) are located in exons (blue boxes with Arabic numerals). Introns are represented as orange boxes. Arrows indicate the direction of new DNA strand synthesis for each primer.
**Figure S2.** (A) PCR amplifications using indicated synthetic dsDNA templates (T) using indicated *TRBC* isoform specific primer pairs (P). No template control (NTC) amplification was included to control for template contamination. No cross amplifications were visible. (B) GAPDH amplification as a DNA template integrity control, showing comparable template load and quality in every sample.
**Figure S3.** Real‐time PCR (qPCR) analysis of TRBC isoform‐specific primer pair specificity. The TRBC1 template DNA was exclusively amplified with the UTRC1 primer pair (specific for TRBC1) and not with UTRC2 (specific for TRBC2). Conversely, the TRBC2 template DNA was only amplified with the UTRC2 primer pair and not with UTRC1, confirming the specificity of each primer set.

## Data Availability

The data that support the findings of this study are openly available in GeneBank at https://www.ncbi.nlm.nih.gov/genbank/, reference number PRJNA1098592.
