# Supplementary material for: Sequence Diversity and Expression Profiles of T Cell Receptor Beta Chain Constant Genes TRBC1 and TRBC2 in Canine Lymphoid Tumour Cell Lines and Normal Lymphocytes
Source: Vet Comp Oncol. 2025 Jul 19;23(4):650–5. doi: 10.1111/vco.70003 (PMC12617685; doi:10.1111/vco.70003)

**Supplementary Table 1** Primers used for PCR and 5’RACE

| primer name | DNA sequence 5’🡪3’ | purpose |
| --- | --- | --- |
| cTRBC_F | AGAGGATCTGCAGAAGGTCA | PCR/qPCR |
| UTRC1 | GCTCTGCCTCTGAACTAGTT | PCR/qPCR |
| UTRC2 | GGCTCATGATCATCTCAGGA | PCR/qPCR |
| GAPDH_F | TGCACCACCAACTGCTTGGC | PCR/qPCR |
| GAPDH_R | GAAGTCACAGGAGACCACCTGG | PCR/qPCR |
| TCR_RACE_F | TCAACCGAGTTTTTTTTTTTTv | 5' RACE |
| TCR_RACE_GS_1 | GATGGTTCAAACACTGTGACC | 5' RACE |
| TCR_RACE_GS_2 | GTGATGGGTTTGACTCTATCGT | 5' RACE |
| TCR_RACE_GS_3 | TTCAGGAACCCTTTCTCTTGAC | 5' RACE |
| 5’-Adaptor_F | GAACTGAACCATTTCAACCGAG | 5' RACE |

| synthetic sequence name | DNA sequence 5’🡪3’ | Length [bp] |
| --- | --- | --- |
| TRBC1 | **cTRBC_F** 🡺  GCGGATCCAGGATCTGCAGAAGGTCACCCCTCCCACGGTCACAGTGTTTGAACCATCAGAAGCAGAGATCTCGCGGACCCAGAAGGCCACGCTCGTGTGCCTGGCCACGGGCTTCTACCCCGACCACGTGGAGCTGAGCTGGTGGGTGAACGGGAAGGAGGTCACGAGTGGGTTCAGCACCGACCCGCAGCCCTACAAGGAGAGGCCCAGCGAGAATGACTCCAGCTACTGTCTGAGCAGCCGGCTGAGGGTCTCTGCCTCCTTCTGGCACAACCCGCGCAACCACTTCCGCTGCCAAGTCCAGTTCTATGGGCTCGGGGACGACGATGAGTGGAAATACGATAGAGTCAAACCCATCACCCAGAACATCAGTGCTGAGGCCTGGGGCAGAGCAGACTGTGGCTTCACCTC  🡸**GS2**  GGTGTCCTACCATCAGGGCGTCCTGTCTGCCACCATCCTCTATGAGATCCTGCTGGGCAAGGCCACGCTGTATGCTGTGCTGGTCAGCGTCCTGGTGCTGATGGTCAAGAGAAAGGGTTCCTGAAACTAGTTCAGAGGCAGAGCCAGCAGCTTCCAGCCTGCTGTTGCCCCAGGACGGATTCTCCTTTTTCCACTTCGGATCCGC  🡸**UTRC1** | 616 |
| TRBC2 | **cTRBC_F** 🡺  AGAGGATCTGCAGAAGGTCACCCCTCCCACGGTCACAGTGTTTGAACCATCAGAAGCAGAGATCTCGCGGACCCAGAAGGCCACGCTCGTGTGCCTGGCCACGGGCTTCTACCCCGACCACGTGGAGCTGAGCTGGTGGGTGAACGGGAAGGAGGTCACGAGTGGGTTCAGCACCGACCCGCAGCCCTACAAGGAGAGGCCCAGCGAGAATGACTCCAGCTACTGTCTGAGCAGCCGGCTGAGGGTCTCTGCCTCCTTCTGGCACAACCCGCGCAACCACTTCCGCTGCCAAGTCCAGTTCTATGGGCTCGGGGACGACGATGAGTGGAAATACGATAGAGTCAAACCCATCACCCAGAACATCAGTGCTGAGGCCTGGGGCAGAGCAGACTGTGGCTTCACCTCGGTGTC  🡸**GS2**  CTACCATCAGGGCGTCCTGTCTGCCACCATCCTCTATGAGATCCTGCTGGGCAAGGCCACGCTGTATGCTGTGCTGGTCAGCATCCTGGTGCTGATGGCCAAGGTCAAGAGAAAAGGTTCCTGAGACCAGCTCCAAAAGTGCATCCTGAGATGATCATGAGCCTCAC  🡸**UTRC1** | 578 |

**Supplementary Table 2** Sequences of synthetic *TRBC1* and *TRBC2* DNA fragments with their length and exact positions of cTRBC_F, GS2, UTRC1 and UTRC2 primers.

**Supplementary Table 3** 5’UTR sequences (grey color), V (variable), D (diversity) and J (joining) regions (green color), and first coding exon of TRBC gene (blue color)

| cell line | 5’🡪3’ DNA sequence | rearrangement type | length [bp] |
| --- | --- | --- | --- |
| CLB70 | CAGTTGCTTCACGAGCCTATTTCTCTCTGTGTGTATATCTACCTCTCTCTCTCTCACACACACACACACACACACACACAGAGCAAACCCAGACAGCTGTCAGCCCAGCCCTGGTCCCAACCTCTCTTCCCTTGCAGAGGATCTGCAGAAGGTCACCCCTC | germline | 161 |
| CNK89 | TGAGGAAGAAAAAAACAGATAGACTCTTGATAGATATGGGCCA  AGGCCTTGAGAAAGAGACTGTCAAGCAAGCCAAGGAAATCAGGACACCCTGCTGCCTTAGGGAGATGGAAGAGATAGTTGGCAGTATGCGCCCATGGGGTAAGCGAGAAAAGTGGCAAAGCTGAGGGAGACTAGAACACGACGGAGACCCCAAACACACAGGACCTACTACTACATAATTTTGCTTTATGATGACTCCATTTCCAGCTCATTCTCTCTCTGTCTCCCTATTTCTCTCTGTTTCTATCTCTGTTCTCTCTCTCTCTCTCTCTCTCTCTCTCTCACACACACACACACACACACACACAGAGCAAACCCAGACAGCTGTCAGCCCAGCCCTGGTCCCAACCTCTCTTCCCTTGCAGAGGATCTGCAGAAG | germline | 421 |
| GL1 | CACACACACACACAGAGCAAACCCAGACAGCTGTCAGCCCAGCC  CTGGTCCCAACCTCTCTTCCCTTGCAGAGGATCTGCAGAAGGTCA  CCCCTC | germline | 95 |
| PER-VAS | CCGTATCCTGCTGTAACATTGTGGGAACTGGGGGCCAATCGGGC  TACGAGCAGTATTTCGGCGCCGGCACCAGGCTCACGAGGATCTG | D1J6 | 88 |
| PER-VAS | GCGATAAGTGGCCAGCAAGAGCCTGCCATCTGGCACTGGTCTGAATTCTTCTCCTTTCCTTCTCAGCACCCCTGGACACAACAGTTTCCCAGACTCCAAGATACCTCATCGCGCACGTGGGATCGAAGAAGTTACTAAAATGTGAGCAAAATCTGGGCCATAATGCTATGTACTGGTATAAGCAAGACCTCAAGCAACTGCTGAAGATCATGTTTATCTACTTTAATCAGGGACTCAATCTAAATGAATCAGTTCCAGGTCGTTTCTCACCTGAGACATCTGACAAAGCTCATTTAAACCTTCATGTCGACTCCCTGGAGACAGGTGACTCTGCTGTGTATTTCTGTGCCAGCAGCCTAGTTTCATCTGGGGAAAGCCAGTCGACGGTCAGTATTTCGGCGGGGGCAAACCTCTCTTCCCTTGCAGAGGATCTGCAGAGACCCACCCT | V3J3 | 448 |

**Supplementary Figure 1**


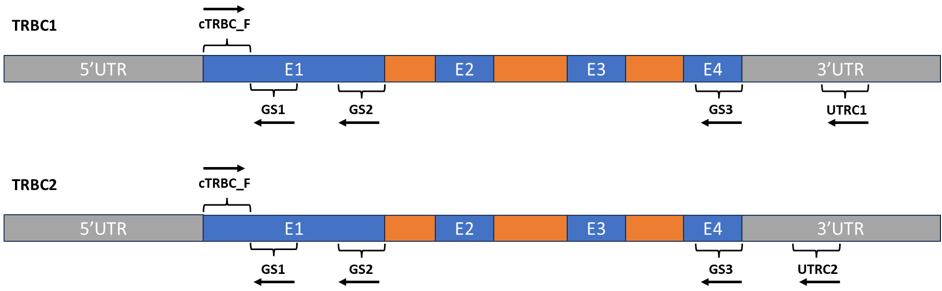


**Supplementary Figure 2**


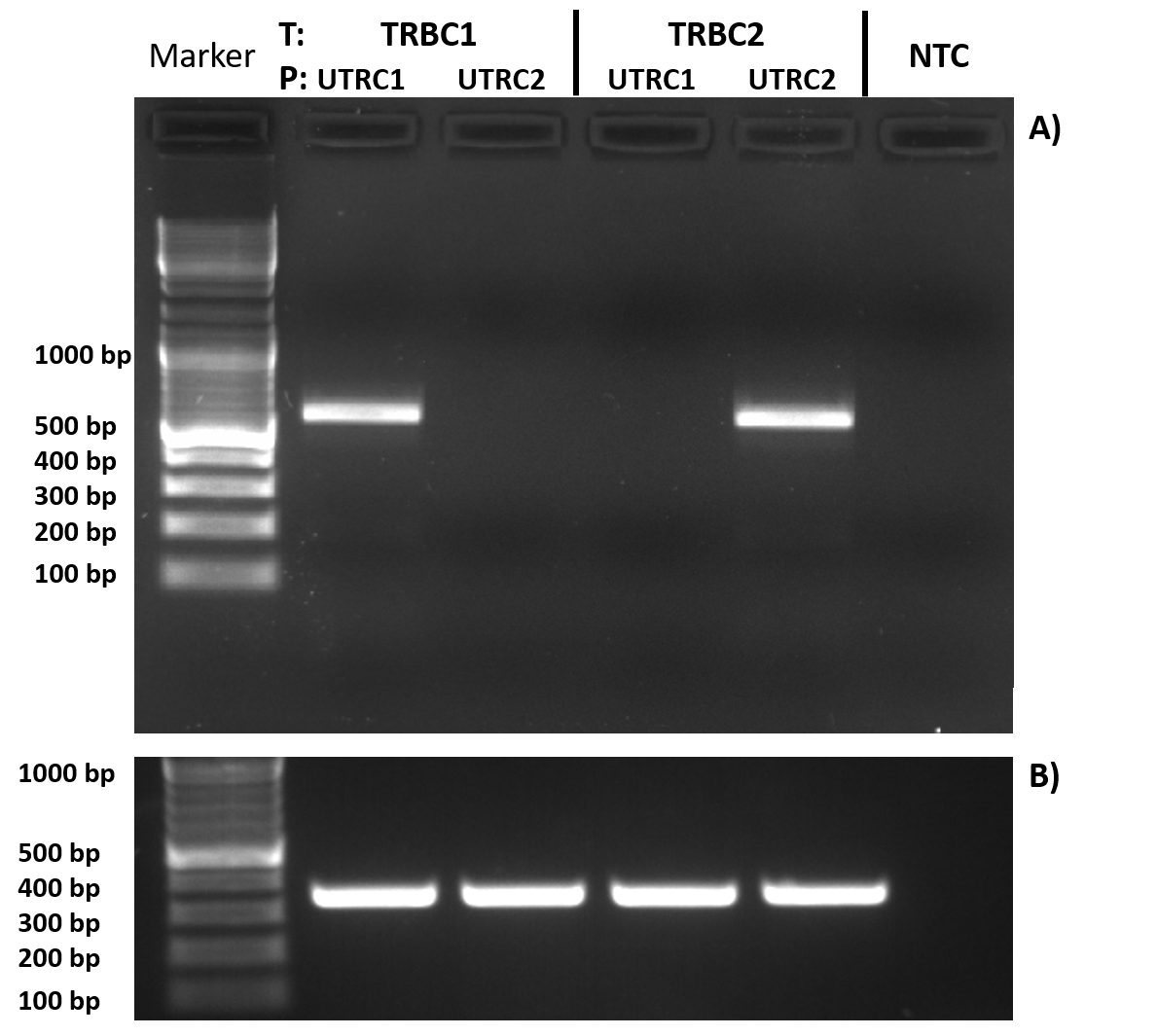


**Supplementary Figure 3**


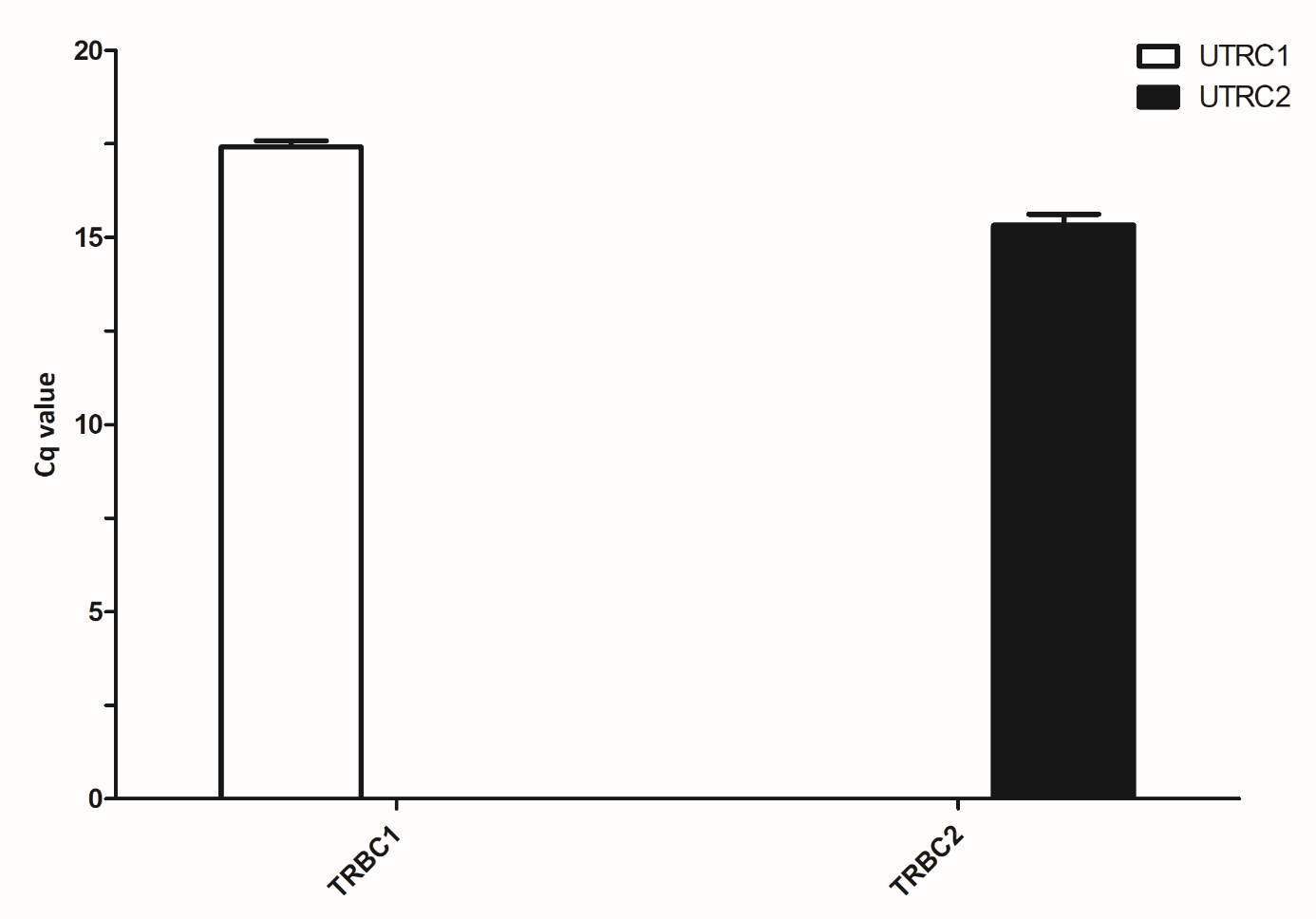

Supplement: Supplementary file 1 — Table S1. Primers used for PCR and 5′RACE. Table S2. Sequences of synthetic TRBC1 and TRBC2 DNA fragments with their length. Table S3. 5′UTR sequences (grey colour), V (variable), D (diversity) and J (joining) regions (green colour), and first coding exon of TRBC gene (blue colour). Figure S1. Schematic representation of the TRBC1 and TRBC2 loci with positions of PCR primers. Gene‐specific primers (UTRC1 and UTRC2) are positioned in 3′untranslated regions (grey boxes) whereas primers common for both TRBC1 and TRBC2 isoforms (cTRBC_F, GS1, GS2, and GS3) are located in exons (blue boxes with Arabic numerals). Introns are represented as orange boxes. Arrows indicate the direction of new DNA strand synthesis for each primer. Figure S2. (A) PCR amplifications using indicated synthetic dsDNA templates (T) using indicated TRBC isoform specific primer pairs (P). No template control (NTC) amplification was included to control for template contamination. No cross amplifications were visible. (B) GAPDH amplification as a DNA template integrity control, showing comparable template load and quality in every sample. Figure S3. Real‐time PCR (qPCR) analysis of TRBC isoform‐specific primer pair specificity. The TRBC1 template DNA was exclusively amplified with the UTRC1 primer pair (specific for TRBC1) and not with UTRC2 (specific for TRBC2). Conversely, the TRBC2 template DNA was only amplified with the UTRC2 primer pair and not with UTRC1, confirming the specificity of each primer set. [file VCO-23-650-s001.docx]
